# More to Explore: Further Definition of Risk Factors for COPD – Differential Gender Difference, Modest Elevation in PM_2_._5_, and e-Cigarette Use

**DOI:** 10.3389/fphys.2021.669152

**Published:** 2021-05-05

**Authors:** Yixuan Zhang, Lu Wang, Gökhan M. Mutlu, Hua Cai

**Affiliations:** ^1^Division of Molecular Medicine, Department of Anesthesiology, Division of Cardiology, Department of Medicine, David Geffen School of Medicine, University of California, Los Angeles, Los Angeles, CA, United States; ^2^Department of Pulmonary and Critical Care Medicine, The First Affiliated Hospital of Zhejiang Chinese Medical University, Hangzhou, China; ^3^Section of Pulmonary and Critical Care Medicine, Department of Medicine, University of Chicago, Chicago, IL, United States

**Keywords:** Chronic obstructive pulmonary disease (COPD), risk factors, cigarette smoking, environmental pollution, gender difference, modest elevation in PM2.5, e-cigarettes

## Abstract

Chronic obstructive pulmonary disease (COPD) is a severe respiratory disease with high morbidity and mortality, representing the third leading cause of death worldwide. Traditional risk factors for COPD include aging, genetic predisposition, cigarette smoking, exposure to environmental pollutes, occupational exposure, and individual or parental respiratory disease history. In addition, latest studies have revealed novel and emerging risk factors. In this review, differential gender difference as a factor for COPD development at different territories is discussed for the first time. First, women seem to have more COPD, while more women die of COPD or have more severe COPD, in Western societies. This seems different from the impression that COPD dominants in men, which is true in Eastern societies. It might be related to higher rate of cigarette smoking in women in developed countries (i.e., 12.0% of women in United States smoke vs. 2.2% in China). Nonetheless, women in Eastern societies are exposed to more biomass usage. Second, modest elevation in PM_2_._5_ levels at >∼21.4–32.7 μg/m^3^, previously considered “cleaner air,” is associated with incidence of COPD, indicating that more stringent goals should be set for the reduction of PM_2_._5_ levels to prevent COPD development. Last but not least, e-cigarette use, which has become an epidemic especially among adolescents as officially declared by the United States government, has severe adverse effects that may cause development of COPD early in life. Built upon an overview of the established risk factors for COPD primarily focusing on cigarette smoking and environmental pollutions, the present review further discusses novel concepts, mechanisms, and solutions evolved around the emerging risk factors for COPD discussed above, understanding of which would likely enable better intervention of this devastating disease.

## Introduction

Chronic obstructive pulmonary disease (COPD) is a prevalent disease characterized by progressive airflow limitation, pulmonary structural abnormalities, and chronic respiratory symptoms including chronic cough, increased sputum production, dyspnea, and wheezing (Celli and Wedzicha, 2019). COPD is the third leading cause of death worldwide according to the World Health Organization (WHO), accounting for 3.0 million deaths in 2016 (World Health Organization [WHO], 2019d). The global prevalence of COPD increased by 44.2% from 1990 to 2015 (GBD 2015 Chronic Respiratory Disease Collaborators, 2017). In the year of 2016, 251 million cases of COPD was identified by the Global Burden of Diseases Study (World Health Organization [WHO], 2019a). The prevalence of COPD in the WHO European countries was up to 4.7% in 2016^1^, whereas in the United States, 15.5 million (5.9%) were diagnosed with COPD in 2015 (Croft et al., 2018). In Asian countries, the prevalence of COPD in India in 2016 was 4.2%, and COPD contributed to 6.4% of the total deaths (Salvi et al., 2018). In China, the China Pulmonary Health study (CPH) reported an overall 8.6% prevalence rate of COPD in a large cohort of 50,991 randomly recruited subjects (between 2012 and 2015) (Wang C. et al., 2018). Despite these mounting data indicating high morbidity and mortality of COPD worldwide, the underlying molecular mechanisms and risk factor profiles of COPD have remained incompletely understood. In the current review, we will discuss established and emerging risk factors of COPD that have been either well studied and acknowledged (cigarette smoking and environmental pollutions/exposures), or noted only recently (territory-dependent differential gender difference, modest elevation in PM_2_._5_ levels, and e-cigarette use). With particular focus on the emerging risk factors, we will discuss concepts, mechanisms, and solutions evolved around these emerging risk factors for COPD, understanding of which would no doubt enable better management of this devastating disease.

## Well-Established Risk Factors

### Cigarette Smoking

During the first decade of the 20th century, cigarette smoking became increasingly popular, first among men and later among women, with increased incidence of chronic bronchitis and lung cancer. In 1964, on the basis of more than 7,000 articles related to smoking and disease, the Advisory Committee to the United States Surgeon General concluded that cigarette smoking is the most important cause of chronic bronchitis^2^. Early in 1970s, researchers started to recognize that cigarette smoking could induce decline in pulmonary function in adults (Cohen, 1978). In a survey in the West Indies and United Kingdom, researchers found that besides symptoms such as coughing and phlegm, cigarette smoking was closely associated with reduction in forced expiratory volume in 1 second (FEV1) (Miller, 1974). A seminal study by Fletcher and colleagues demonstrated that cigarette smoking can lead to airflow limitation (declined FEV1) and consequent COPD (Fletcher, 1976; Fletcher and Peto, 1977). Later, the Lung Health Study (LHS) was conducted in 1986–1994 (with a long-term follow-up of 14.5 years), data from which demonstrated that tobacco cessation significantly improved FEV1 in smokers with mild obstructive pulmonary disease and reduced COPD morbidity and mortality (Anthonisen et al., 1994, 2005). Of note, smoking cessation improved FEV1 in COPD patients as early as 6 weeks post-cessation (Dhariwal et al., 2014). To date, accumulating data have indicated a dose-dependent association between cigarette smoking and COPD. Obstruction of the airway becomes worse with increasing mean pack years smoked (Burney et al., 2014). Recently, a cross-sectional study of a large multicenter cohort (COPDGene) of current and former smokers has shown that smoking duration was negatively associated with adjusted means of FEV1/FVC ratio (Bhatt et al., 2018). The recent CPH study also demonstrated a dose-dependent association between cigarette smoking and a decline in pulmonary function (Wang C. et al., 2018).

Of note, early life exposure to cigarette smoking predicts development of COPD in adults (The Lancet Respiratory Medicine, 2020). As described in a systematic review covering 16 studies of 69,365 individuals, tobacco exposure *in utero* and early life represents an important risk factor for development of COPD in adulthood (Savran and Ulrik, 2018). In addition, among ever smokers, people with an early age of smoking debut appeared to be more vulnerable to pulmonary respiratory symptoms (Erbas et al., 2018). Cigarette smoking has also been shown to augment the adverse effects of early life exposures (infant respiratory infection and home overcrowding) (Allinson et al., 2017) on mid life FEV1 and FVC.

To date, multifaceted mechanisms of oxidative stress, inflammation, and tissue destruction have been implicated in the pathogenesis of COPD (Stevenson et al., 2006; Chung and Adcock, 2008; Zuo et al., 2014). In the setting of cigarette smoking, smokers are exposed to more than 7,000 components in its gaseous and particulate phases, including nicotine, direct carcinogens (such as acrolein, benzo-pyrenes, methylcholanthrene, and heavy metals), toxins (such as acetone, ammonia, carbon monoxide, and hydroquinone), oxidants (such as nitrogen oxides and superoxide), and reactive solids with chemically catalytic surfaces (Staempfli and Anderson, 2009; U.S. Department of Health and Human Services, 2010; Ganguly et al., 2018). In general, exposure to cigarette smoking induces inflammation through increased production of cytokines and/or chemokines, which subsequently recruits neutrophils, macrophages, and dendritic cells to exacerbate inflammation. The recruitment of inflammatory cells leads to reactive oxygen species (ROS) production and surface antigen formation-mediated innate and adaptive immune responses. Of note, oxidative stress plays a central role in activating several pathogenic events. ROS, generated from inflammatory responses or present in the cigarette smoke, cause DNA damage and gene expression of proinflammatory mediators (MacNee and Rahman, 1999; Cai and Wang, 2017b). Oxidative stress also results in airspace epithelial injury (MacNee and Rahman, 1999). In addition, ROS production increases the expression and activity of metalloproteinases and, at the same time, inactivates antiproteinases, leading to degradation of matrix proteins (MacNee and Rahman, 1999; Seagrave, 2000). More importantly, ROS production is believed to initiate ROS-induced ROS release and thus maintain sustained oxidative stress (Cai and Wang, 2017b), representing a central pathway mediating pathological processes of COPD.

According to the official American Thoracic Society policy statement in 2010, the estimated population-attributable fraction (PAF) of smoking for COPD was almost 80% in most countries/areas (Eisner et al., 2010). Of note, PAF is used to show the contribution of a risk factor to a disease or a death. Due to the fact that many diseases are caused by multiple risk factors, which may also interact with each other, the total of PAFs for individual risk factors may be more than 100% in some cases (World Health Organization [WHO], 2020). Nonetheless, the PAF values are at least in part reflective of contribution of individual risk factors. On the other hand, an estimated 15–45% of patients with COPD have never smoked, according to current literatures (Salvi and Barnes, 2009; Lamprecht et al., 2011; Thomsen et al., 2013; Tan et al., 2015; Rajendra et al., 2018; Syamlal et al., 2019). Therefore, genetic factors such as those can be identified through family history, and other risk factors, including environment pollutions (household and ambient air pollution) and/or epigenetic regulations in response to environmental pollutions, may play important roles in the pathogenesis of COPD in the never-smokers.

### Environmental Pollutions

Environmental exposure (including ambient/outdoor and household air pollutions) is a non-selective and unavoidable traditional risk factor for COPD that has synergistic effects with cigarette smoking in inducing COPD-related morbidity and mortality (Cai and Wang, 2017b; Rajendra et al., 2018). It has been shown that ambient (outdoor) air pollution induces pathogenesis of COPD through PM_2_._5_ (PMs with diameter of 2.5 μm or less) and PM_10_ (PMs with a diameter of 10 μm or less) (Guan et al., 2016; Doiron et al., 2019). Accumulating evidence has indicated that ambient levels of these particles have a dose-time relationship with incidence and mortality of COPD. In the CPH study, the authors found that exposure to a higher level of PM_2_._5_ (annual mean exposure ≥75 μg/m^3^) was significantly associated with higher prevalence of COPD among never-smokers (Wang C. et al., 2018). In addition, it has been established that PM_2_._5_ concentrations were significantly associated with increased risk of hospital administration of COPD (Atkinson et al., 2014).

Meanwhile, occupational exposures are important, intervenable causes of COPD, which also represent one particular type of outdoor air pollution (Eisner et al., 2010; Syamlal et al., 2019). According to the report of the American Thoracic Society, occupational exposure contributes to 15–20% of COPD risk (Eisner et al., 2010). For COPD patients who have never smoked, 26–53% can be attributed to workplace exposures, including dust, fumes, gases, vapors, and secondhand smoke exposures (Syamlal et al., 2019). The results of the Swiss Cohort Study on Air Pollution and Lung and Heart Diseases in Adults indicate significant association of high levels of occupational exposures with increased incidence of COPD (Mehta et al., 2012). Approximately 10–15% increase in risk of stage II COPD was reported for every 10 years of cumulative workplace exposure (Mehta et al., 2012). It has been reported that six occupations under United Kingdom Standard Occupational Classification (SOC) are associated with increased COPD risk among never-smokers and never-asthmatics, including “sculptor, painter, engraver, art restorer” (SOC code 3411); “gardener, groundsman, park keeper” (SOC code 5113); “food, drink, and tobacco processor” (SOC code 8111); “plastics processor, molder” (SOC code 8116); “agriculture, and fishing occupations not elsewhere classified” (SOC code 9119); and “warehouse stock handler, stacker” (SOC code 9251) (De Matteis et al., 2019). Therefore, job history needs to be evaluated for COPD risk and incidence, especially for never-smokers (Syamlal et al., 2019). About 16% of coal workers developed pneumoconiosis, an occupational disease, due to accumulation of inhaled coal mine dust in the lung (American Lung Association, 2020). Coal mine dust comprises carbon, quartz, and silicates (Blanc and Seaton, 2016). It has been shown that coal mine dust induces inflammatory response in the lung and leads to silicosis and emphysema (Blanc and Seaton, 2016).

Household air pollution is generated from cooking and heating using biomass and coal, collectively known as solid fuels. According to WHO, about three million people are exposed daily to smoke from insufficient combustion of solid fuels around the world, especially in less developed countries. Accumulating evidence indicates that household air pollution exposure is associated with higher prevalence of COPD in low- and middle-income countries/areas (Salvi and Barnes, 2009; van Gemert et al., 2015; Magitta et al., 2018; Siddharthan et al., 2018). Based on a more recent prospective cohort study from 9,835 COPD cases with a 9.1-year follow-up in the China Kadoorie Biobank, using coal and wood burning for cooking was positively associated with the incidence of COPD (Li et al., 2019). Of note, COPD risk increases with the number of years of using solid fuel for heating and wood for cooking (Li et al., 2019).

Studies have shown that biomass smoke exerts a deteriorating health impact mainly through deposition in the airway mucosa of particulate matter, nitrogen dioxide, carbon monoxide, sulfur oxide, formaldehyde, and polycyclic organic matter (Bruce et al., 2000; Salvi and Barnes, 2009). These substances have strong oxidant properties that may cause chronic inflammation and structural changes in the airways and alveoli (Salvi and Barnes, 2010). Amongst the substances in biomass smoke, PM_10_ was shown to have major adverse health effects (Salvi and Barnes, 2010). The WHO air quality guideline value for PM_10_ concentration is 50 μg/m^3^ in 24 h^3^. However, previous studies have shown that the mean 24 h PM_10_ levels are about 300–3,000 μg/m^3^ in homes that use biomass fuels, and may reach 30,000 μg/m^3^ during cooking (Bruce et al., 2000; Salvi and Barnes, 2009; Eisner et al., 2010; Kurmi et al., 2012). Moreover, the WHO air quality guideline value for PM_2_._5_ is 25 μg/m^3^ in 24 h. However, the average of 24 h PM_2_._5_ levels was reported to range from 490 to 1,400 μg/m^3^ in homes using biomass fuels, and the maximum levels may exceed 70,000 μg/m^3^ during cooking (Kurmi et al., 2012; Chen et al., 2016; Li et al., 2016; Elf et al., 2018; Julia Jung, 2018). Similar to cigarette smoking, biomass smoke exposure also has dose-response effects on airway obstruction, while early onset and longer time of exposure are associated with increased risk of COPD (Kurmi et al., 2012).

## Emerging Novel Risk Factors for COPD

Importantly, recent studies have revealed novel risk factors for COPD that have not been previously recognized, including but not limited to differential gender difference in different territories, modest elevation in PM_2.5_ levels, and use of e-cigarettes/vaping devices. These emerging risk factors for COPD are discussed in details below.

### Differential Gender Difference

The prevalence of COPD in the United States has increased more in women than in men in the recent years (Han et al., 2007; Akinbami and Liu, 2011). Women are 37% more likely to have COPD than men after adjustment for age, smoking status, and ethnicity (Pinkerton et al., 2015). Women also have higher risk of hospitalization for COPD than men (Prescott et al., 1997). Moreover, more women than men have died of COPD in the United States since 2000, whereas more men have died of COPD prior to 2000 (Han et al., 2007). Of note, women account for 53% of all deaths from COPD in United States (Pinkerton et al., 2015). In addition, it has been shown that the prevalence of COPD (in patients ≥40 years old) increased in women and decreased in men in Netherlands during the period of 1980–2006 (Bischoff et al., 2009).

However, in developing countries such as China and India, the prevalence and mortality of COPD in women is substantially lower than that in men (Salvi et al., 2018; Wang C. et al., 2018; Zhu et al., 2019). It has been reported that men had a higher prevalence (11.9%) of COPD than did women (5.4%) in China between June 2012 and May 2015 (Wang C. et al., 2018). The estimated number of men with COPD in 2015 was 68.4 million, while the number was 31.5 million for women (Wang C. et al., 2018). Accordingly, the mortality rate of COPD in men aged 45–64 was 22.91 (per 100,000 per year) in 2016, and the number was 10.26 for women (Zhu et al., 2019). After age of 65, the mortality rates are 568.76 in men and 403.71 in women (per 100,000 per year) (Zhu et al., 2019). In India, the prevalence of COPD at the age of 45–49 was 7.3% in men and 4.8% in women, whereas it was 28.6% in men and 17.6% in women at the age of 65–69 (Salvi et al., 2018).

Therefore, there seems to be a clear differential trend of gender difference in COPD mobility between the developed and the developing countries (Bischoff et al., 2009; U.S. Department of Health Human Services et al., 2011; Pinkerton et al., 2015; Salvi et al., 2018; Wang C. et al., 2018; Zhu et al., 2019). This might be at least partially attributed to distinct prevalence of cigarette smoking in these territories. According to the 2018 National Health Interview Survey (NHIS), 12.0% of females and 15.6% of males were current cigarette smokers in adults aged ≥18 years in the United States (Creamer et al., 2019), although the prevalence of smoking in United States adults has dropped dramatically in the past 50 years (42.4% in 1965, vs. 14% in 2017) (Wang T.W. et al., 2018). On the other hand, 2.2% of women and 49.8% of men smoked between 2012 and 2015 in China (Wang C. et al., 2018). A similar trend was reported in 2010 in India, where prevalence of cigarette smoking or bidis (locally manufactured tobacco) smoking is much less in women than in men (2.7% in women vs. 24% in men, 11 vs. 108 million) in adults (15–69 years old) (Mishra et al., 2016). These data suggest that lower COPD prevalence in women than in men in these territories might be attributed to fewer cigarette smokers in women, indicating an important role of cigarette smoking in largely dictating the development of COPD in different gender groups, and the importance of further augmenting smoking cessation in the prevention of the disease.

As we have discussed above, exposure to household air pollution is associated with increased prevalence and mortality of COPD (Salvi et al., 2018; Siddharthan et al., 2018; Wang C. et al., 2018; Chan et al., 2019), and is considered one of the primary risk factors for non-smoking-related COPD especially in low- and middle-income countries (Gordon et al., 2014; Gut-Gobert et al., 2019; World Health Organization [WHO], 2019b). The risk of COPD nearly doubles (in men) or is more than doubled (in women) in population exposed to high levels of household air pollution than those who use cleaner fuels and technologies (World Health Organization [WHO], 2019b). Of note, in many cultures women are mostly responsible for domestic cooking. As a result, women are exposed to high levels of household air pollution more frequently than men (Gordon et al., 2014). Indeed, it has been shown that the association between COPD and household air pollution exposure was stronger in women than in men in 13 low- and middle-income country settings (Latin America, sub-Saharan Africa, and Southeast Asia) (Siddharthan et al., 2018). Especially, household air pollution contributed to COPD mortality at 22.7% in men and 30.1% in women in India (Salvi et al., 2018). It is noteworthy that women living in severe poverty have the greatest exposure to household air pollution (Gordon et al., 2014; World Health Organization [WHO], 2019c). Nonetheless, since women from these territories still have lower COPD prevalence, it seems to suggest that cigarette smoking is more detrimental than biomass exposure in contributing to the etiology of COPD.

### Modest Elevation in PM_2.5_ Levels

As discussed above, exposure to high level of PM_2_._5_ is associated with development of COPD. However, modest elevation in PM_2_._5_ levels is not harmless. Growing evidence has suggested that exposure to modestly elevated levels of PM_2_._5_ may also induce or accelerate development of COPD. A study of participants aged 20 years or older has reported that exposure to PM_2_._5_ at the concentrations of 21.42–23.94, 23.94–31.86, or higher than 31.86 μg/m^3^ significantly increased development of COPD (vs. <21.42 μg/m^3^) (Guo et al., 2018). Another study conducted in adult non-smokers showed similar results (Huang et al., 2019). It is reported that exposure to PM_2_._5_ at concentrations higher than 38.98 μg/m^3^ is associated with development of COPD (Huang et al., 2019). A study of 5,993 residents (aged of 20 or above) from Guangdong, China, has reported that a higher year-round daily mean of PM_2_._5_ (more than 35 μg/m^3^) is associated with higher COPD prevalence (Liu et al., 2017). In addition, the recent CPH study of 50,991 Chinese participants (aged 20 years or older) indicated that an annual mean exposure to PM_2_._5_ between 50 and 74 μg/m^3^ is associated with higher prevalence of COPD in never-smokers (vs. <50 μg/m^3^) (Wang C. et al., 2018). The association between modestly elevated PM_2_._5_ levels and COPD has also been reported in Korea (Lamichhane et al., 2018). Exposure to PM_2_._5_ levels at 32.7–37.1 or higher than 37.1 μg/m^3^ had significantly higher risk of developing COPD in the age group of 20–85 years old (vs. <32.7 μg/m^3^) (Lamichhane et al., 2018). These data implicate a novel observation that modestly elevated levels of PM_2_._5_ are associated with increased risk of developing COPD in adults.

The underlying mechanisms of PM_2_._5_-associated COPD involve induction of oxidative stress and inflammation. It has been reported that exposure to PM_2_._5_ enhances pulmonary oxidative stress (Deng et al., 2013; Jin et al., 2018), which is consequent to the Fenton reaction of the transition metals coated on PM_2_._5_ (Cho et al., 2018). In addition, exposure to PM_2_._5_ induces pulmonary inflammation and impairs lung function (Zhao et al., 2012, 2019; He et al., 2017). PM_2_._5_-induced ROS upregulates expression of pro-inflammatory factors, such as TNF-α, IL-6, IL8, and MCP-1 (He et al., 2017; Cho et al., 2018; Lin et al., 2018). Treatment with antioxidant TEMPOL or NAC prevented PM_2_._5_-induced inflammation in mouse aortas or lungs (Haberzettl et al., 2016; Liu et al., 2018). It has also been shown that PM_2_._5_ induces ROS-dependent autophagy of pulmonary macrophages in rats (Su et al., 2017). Previous studies suggest that STAT, NF-κB, Wnt, ERK, p38, and PI3K/Akt/mTOR signaling are involved in PM_2_._5_-induced inflammatory responses in mouse models (Su et al., 2017; Cho et al., 2018; Liu et al., 2018; Wang et al., 2019). However, the mechanisms underlying increased risk of developing COPD when exposed to modestly elevated levels of PM_2_._5_ remain to be further investigated, for example, if lower levels of ROS and/or alternative pathways are involved.

### Use of Electronic Cigarettes

Electronic cigarettes (e-cigarettes) are battery-operated electronic nicotine delivery systems (or “devices”). An e-cigarette contains a mouthpiece (to inhale), a power source, a heating element (atomizer), and a disposable cartridge or refillable tank with liquid solution (e-liquid) (Bhatnagar et al., 2014; Cai and Wang, 2017a; Centers for Disease Control and Prevention [CDC], 2019a; Layden et al., 2019; National Institute on Drug Abuse, 2019). The e-liquid contains propylene glycol, glycerin, nicotine, and flavor chemicals (Bhatnagar et al., 2014; Cai and Wang, 2017a). Upon puffing-activated heating, the e-liquid is atomized, and the smoker inhales the resulting aerosol or vapor.

#### High Prevalence of e-Cigarette Use, Especially in Youth

The use of e-cigarettes has been increasingly mounting. The prevalence of ever use of e-cigarettes in adults in the United States drastically increased within last decade (1% in 2009 vs. 14.9% in 2018) (Pepper and Brewer, 2014; Villarroel and Vahratian, 2020). More than half of the current and ex-smokers have ever used e-cigarettes in Great Britain, 20% of whom are currently using e-cigarettes (Brown et al., 2014). Till 2015, the percentage of urban current and ex-smokers who had tried e-cigarette has increased 5.5-fold in China since 2009 (2% in 2009 vs. 11% in 2015) (Tobaco Control Resource Center, 2018). The increased use of e-cigarettes may be directly related to the perception of e-cigarettes being less harmful than conventional cigarettes or as tools to quit smoking. However, e-cigarettes are not a United States Food and Drug Administration (FDA)-approved smoking cessation aid. In fact, the United States Preventive Services Task Force has concluded that the current evidence is insufficient to recommend e-cigarette for tobacco cessation in adults (Patnode et al., 2015), although in the United Kingdom, e-cigarettes have been used as prescription smoking-cessation tools (Parlimentary Office of Science and Technology, 2016).

It is noteworthy that the use of e-cigarettes among youth has become a new critical concern for public health. It is worrisome to note that 20.8% of high school students and 4.9% of middle school students used e-cigarettes in the United States in 2018 (Cullen et al., 2018). Nowadays, e-cigarettes have become the most commonly used tobacco product among the students in high school and middle school in United States (Gentzke et al., 2019). It is reported that the prevalence of e-cigarette use among high school students increased more than 13-fold between 2011 and 2018 (Cullen et al., 2018). In the past 2 years, the number almost doubled (11.7% in 2017 vs. 20.8% in 2018) (Cullen et al., 2018) and it continued to rise up to 27.5% in 2019 (Cullen et al., 2019). In February 2019, the FDA announced adolescent use of e-cigarettes an epidemic, and one of the biggest public health challenges (FDA a). Five surveys [the Youth Tobacco Policy Survey, the Schools Health Research Network Wales survey, two Action on Smoking and Health (ASH) Smoke free Great Britain-Youth Surveys, and the Scottish Schools Adolescent Lifestyle and Substance Use Survey] conducted during 2015 and 2016 suggested that 7–32% youth have ever used e-cigarettes in United Kingdom (Bauld et al., 2017; Venkatesan, 2017). Notably, ASH surveys showed a significant rise of ever use of e-cigarettes in youth (7% in 2016 vs. 12% in 2018) (Bauld et al., 2017; ASH, 2020). According to the most recent results of Chinese Youth Tobacco Survey, the prevalence of current e-cigarette user in middle school students was 2.7% in 2019, which is more than doubled when compared to 1.2% in 2014 (Chinese Center for Disease Control and Prevention, 2020).

One of the reasons for the prevalence of e-cigarettes among youth seems to be the desirable flavors (Tsai et al., 2018). Previous studies have shown that flavor was a primary reason for using a tobacco product, especially among teenagers (Villanti et al., 2017). Eighty-one percent of teenagers used flavored product as their first tried tobacco product, whereas the percentage in adults was 54% (Villanti et al., 2017). According to the National Youth Tobacco Survey in 2019, the most popular flavors among high school e-cigarette users are fruit, menthol or mint, and candy (Cullen et al., 2019). Previous studies have shown that some flavoring chemicals induce inflammation in the lung, which will be further discussed below in the Section of “E-Cigarette Use May Predispose to COPD Development.”

#### E-Cigarette Use May Predispose to COPD Development

It has been shown that use of e-cigarettes in youth promotes teenagers’ adaptation to tobacco smoking, a known risk factor for COPD (Schraufnagel et al., 2014; US Department of Health Human Services, 2016). According to reports from United Kingdom, China, and United States, youth who use e-cigarettes are more likely than non-users to try conventional cigarettes subsequently (McNeill et al., 2018; Xiao et al., 2018; Berry et al., 2019). In addition, as we have discussed in the Section of “Cigarette Smoking,” early life exposure to cigarette smoking has been shown to associate with a decline in pulmonary function and development of COPD in adulthood. Therefore, it is predictable that high prevalence of e-cigarette use would promote debut of cigarette smoking in teenagers, which may predeposit vulnerability to pulmonary function and COPD in adulthood. Moreover, exposure to nicotine during adolescence may lead to long-term brain damage and result in adverse effects in neurobiological responses in later life, such as detrimental effects on cognition, attention, and mood (Iniguez et al., 2009; US Department of Health Human Services, 2016). Besides nicotine, the e-liquid contains glycerol, propylene glycol, and flavor chemicals. Though glycerin and propylene glycol are considered non-toxic when delivered orally, acrolein (from glycerol) and propylene oxide (from propylene glycol) are generated during the heating process of e-cigarettes (Cai and Wang, 2017a). Acrolein is a known respiratory toxicant generated by conventional cigarettes, which has been shown to be associated with the development of COPD (Cai and Wang, 2017a; Lin et al., 2019). A recent study has shown that e-cigarettes produce acrolein to a similar level as produced by conventional cigarette (Lin et al., 2019). Treatment of epithelial airway cells with e-cigarette vapor induced dysfunction of ion channels (Lin et al., 2019), which was caused by acrolein-dependent covalent modification of the ion channel protein (Raju et al., 2017). In addition, propylene glycol is not present in traditional cigarettes. Exposure to aerosolized mixture of propylene glycol and glycerol (mimic the solvent of e-liquid without flavors) reduced cell membrane fluidity and impaired protein diffusion in human bronchial epithelial cells, suggesting impaired cell function (Ghosh et al., 2018). This is probably due to production of hydroxyl radicals generated from propylene glycol alone or its combination with glycerol (Son et al., 2019). Of note, increased oxidative stress in the lung contributes to a variety of pathways that mediate development of COPD (Repine et al., 1997).

**FIGURE 1:**
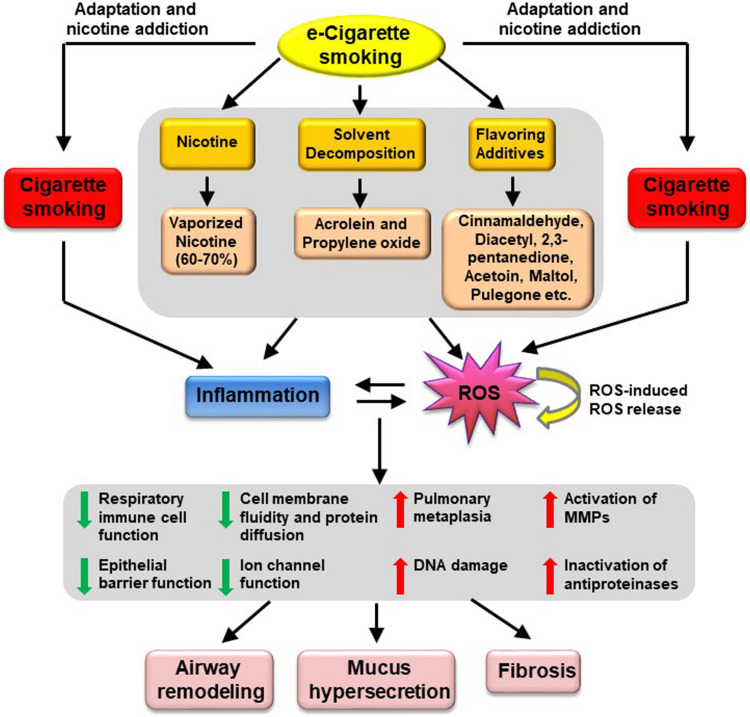
Proposed mechanisms underlying e-Cigarette-induced pathogenesis of COPD. Vaping of e-Cigarettes produces vaporized nicotine and solvent decomposition products (i.e., acrolein and propylene oxide); together with flavoring additives such as cinnamaldehyde, diacetyl, 2,3-pentanedione, acetoin, maltol, pulegone, these products are known to induce oxidative stress and inflammation, which will in turn trigger cellular responses including reduced respiratory immune cell function, impaired epithelial barrier function, reduced cell membrane fluidity and protein diffusion, reduced ion channel function, increased pulmonary metaplasia, increased DNA damage, increased activation of MMPs and increased inactivation of antiproteinases. These pathophysiological alterations may ultimately lead to airway remodeling, mucus hypersecretion, and fibrosis, resulting in development of COPD. MMPs, metalloproteinases.

In addition, some of the flavoring chemicals have been reported to be harmful to cells in the lung including cinnamaldehyde (cinnamon flavor), diacetyl and 2,3-pentanedione or acetoin (butter flavor), maltol (malt flavor), pulegone (menthol and mint flavor) (Lerner et al., 2015; Zaccone et al., 2015; Behar et al., 2016; Clapp et al., 2017; Gerloff et al., 2017; Jabba and Jordt, 2019). Cinnamaldehyde is the major chemical in cinnamon-flavored e-cigarettes, which has been shown to inhibit respiratory immune cell function (Clapp et al., 2017). It also impaired epithelial barrier function in human bronchial epithelial cells (Gerloff et al., 2017). The flavor of cinnamon additives induced inflammatory responses in cultured lung fibroblasts (Lerner et al., 2015). Treatment of airway epithelial cells with butter flavor chemicals (diacetyl and 2,3-pentanedione) reduced Na^+^ transport, an important function of the epithelium in the lung (Zaccone et al., 2015). Another butter flavor chemical acetoin was reported to potently induce proinflammatory response (through induction of IL-8 release) and inhibit epithelial barrier function in human bronchial epithelial cells (Gerloff et al., 2017). Similarly, maltol (malt flavor) induced a proinflammatory response in human lung fibroblasts cells (Gerloff et al., 2017). Pulegone, a chemical for menthol and mint flavors, is a carcinogen that induces pulmonary metaplasia (Jabba and Jordt, 2019). A very recent report suggested that users of menthol and mint-flavored e-cigarettes may be exposed to higher levels of pulegone than that is considered acceptable by the FDA for intake in food, and even higher than in smokers of conventional menthol cigarettes (Jabba and Jordt, 2019). In January 2020, FDA issued an enforcement against unauthorized flavored e-cigarette products (FDA, 2020b). Companies that do not cease manufacture, distribution, and sale of unauthorized flavored cartridge-based e-cigarettes product (other than tobacco- or menthol-flavored) would risk FDA enforcement actions (FDA, 2020b). This enforcement also protects minors from being targeted or promoted to use e-cigarette products (FDA, 2020b). The proposed mechanisms underlying e-cigarette induced pathogenesis of COPD are presented in Figure 1.

Last but not least, e-cigarettes aerosols may contain heavy metals (such as copper, lead, and cadmium), which are known to cause respiratory distress and diseases (US Department of Health Human Services, 2016). It has been reported that copper particles induced ROS production in mitochondria, leading to mitochondrial dysfunction in lung fibroblasts (Lerner et al., 2016). Previous studies also reported that nickel and chromium are present in e-cigarette aerosols, even though they do not exist in the e-liquids (Traboulsi et al., 2020). These may be resulted from heavy metal vaporized into vaping aerosols from the heated coils of e-cigarette, which are usually made with nichrome (combination of nickel and chromium) and stainless steel (Traboulsi et al., 2020).

#### Enforcement on e-Cigarette Regulations

In August 2018, the FDA declared e-cigarette use in youth an epidemic (Cai et al., 2020), and has since taken several immediate actions to establish new policies aimed at preventing youth access to e-cigarettes in the following months (FDA, 2019a, c, d, e). As early as in July 2019, WHO released recommendations on e-cigarettes regarding legislation of e-cigarette control, especially in youth (Cai et al., 2020). The outbreak of e-cigarette or vaping product use associated lung injury (EVALI) last fall in the United States has raised more concerns of e-cigarette use/vaping. The first case of EVALI was reported on August 23, 2019 in Illinois; and since then, EVALI has resulted in 2,807 hospitalization or deaths in all 50 states, the District of Columbia, and two United States territories (as of February 18, 2020) (Centers for Disease Control and Prevention [CDC], 2019b). Sixty-eight deaths have been confirmed with EVALI (as of February 18, 2020) (Centers for Disease Control and Prevention [CDC], 2019b). Fifteen percent of the cases are under 18 years old, and 61% of the cases are 18–34 years old (Centers for Disease Control and Prevention [CDC], 2019b). Approximately 82% of the EVALI patients reported a history of using THC (tetrahydrocannabinol, a component of marijuana)-containing products (Centers for Disease Control and Prevention [CDC], 2019b), although the patients who did not use THC products also displayed similar severe lung injuries. Quickly on September 11, 2019, United States government announced a plan to ban the sales of flavored e-cigarettes and FDA intended to finalize a compliance policy that would prioritize enforcement of premarket authorization requirements for non-tobacco-flavored e-cigarettes (FDA b). This would clear the market of unauthorized, non-tobacco-flavored e-cigarettes, including the most popular flavors among youth – fruit, menthol/mint, and candy (FDA b). Several cities, states, and areas have banned the sales of flavored or all vaping products to fight against e-cigarettes/vaping-related illness, including City of San Francisco, City of Livermore, Michigan State, City of Richmond, New York State, Massachusetts State, Rhode Island, Washington State, Los Angeles County, and Oregon State. The marketing leader of e-cigarettes Juul Labs has agreed to stop all advertising after FDA’s warning and Federal prosecutor’s investigation (FDA, 2019b; npr; The Wall Street Journal, 2019). Major media such as CBS, Warner Media, and Viacom have dropped all e-cigarette advertising. Of note, some large retailers including Rite Aid, Walmart, Kroger, and Walgreens have stopped sales of e-cigarettes. With the same consideration of pulmonary health and to protect youth from becoming addicted to nicotine, Juul sales halted on Chinese websites just days after its launch on September 17, 2019. Around the same time, India has approved an executive order banning e-cigarette products, including manufacture, import and export, sale, distribution, and advertisement. In early November 2019, China banned online advertising and sales of e-cigarettes to protect youth from using e-cigarettes. On January 2, 2020, FDA issued a policy prioritizing enforcement against unauthorized flavored e-cigarette products that appeal to youth (FDA, 2020b). And the guidance document of prioritizing the enforcement was released in April 2020 (FDA, 2020a).

## Conclusion

In summary, we propose that with the recognition of both established and emerging risk factors for COPD, more stringent goals should be set to reduce risk of COPD development by prompting smoking cessation, eliminating all types of environmental pollutions/exposures, strategically preventing declines in pulmonary function in men and women according to their smoking status and indoor environmental exposure, specifically decreasing levels of ambient PM_2_._5_ to the levels below ∼21.4–32.7 μg/m^3^, and limiting the use of e-cigarettes. Since gene-environment-interaction has been implicated in the pathogenesis of COPD (Cai and Wang, 2017b), understanding the potential interactions between individual genetic background and modest elevation in PM_2_._5_/e-cigarette use might provide further insights into the mechanisms of COPD development. Based on the accumulating evidence discussed in the present review, these strategies may prove to be highly beneficial in reducing the risk of COPD development and progression globally in all ethnic groups.

## Author Contributions

YZ and LW drafted and revised the manuscript. GM reviewed and edited the manuscript. HC was responsible for conceptualization, and drafting, revising, and finalizing of the manuscript. All authors contributed to the article and approved the submitted version.

## Conflict of Interest

The authors declare that the research was conducted in the absence of any commercial or financial relationships that could be construed as a potential conflict of interest.

## Footnotes

1 https://gateway.euro.who.int/en/indicators/hfa_403-2510-prevalence-of-chronic-obstructive-pulmonary-disease/

2 https://www.cdc.gov/tobacco/data_statistics/sgr/history/index.htm

3 https://www.who.int/news-room/fact-sheets/detail/ambient-(outdoor)-air-quality-and-health
